# Prognostic value of biomarkers after cardiopulmonary bypass in pediatrics: The prospective PANCAP study

**DOI:** 10.1371/journal.pone.0215690

**Published:** 2019-06-17

**Authors:** Sara Bobillo-Perez, Iolanda Jordan, Patricia Corniero, Monica Balaguer, Anna Sole-Ribalta, Maria Esther Esteban, Elisabeth Esteban, Francisco Jose Cambra

**Affiliations:** 1 Pediatric Intensive Care Unit, Hospital Sant Joan de Déu, Pediatric Intensive Care Research Group, Institut Recerca Hospital Sant Joan de Déu, Universitat de Barcelona, Barcelona, Spain; 2 Pediatric Intensive Care Unit, Sant Joan de Déu Hospital, Pediatric Intensive Care Research Group, Institut Recerca Hospital Sant Joan de Déu, CIBERESP, Barcelona, Spain; 3 Pediatric Intensive Care Unit, Hospital Sant Joan de Déu, Barcelona, Spain; 4 Section of Zoology and Biological Anthropology, Department of Evolutionary Biology, Ecology and Environmental Sciences, Faculty of Biology, Universitat de Barcelona, Barcelona, Spain; 5 Institut de Recerca de la Biodiversitat (IRBio), Universitat de Barcelona, Barcelona, Spain; Ospedale del Cuore G Pasquinucci Fondazione Toscana Gabriele Monasterio di Massa, ITALY

## Abstract

**Objective:**

To assess the usefulness of procalcitonin, pro-adrenomedullin and pro-atrial natriuretic peptide as predictors of need for mechanical ventilation and postoperative complications (need for inotropic support and bacterial infection) in critically ill pediatric patients after cardiopulmonary bypass.

**Design:**

A prospective, observational study

**Setting:**

Pediatric intensive care unit.

**Patients:**

Patients under 18 years old admitted after cardiopulmonary bypass.

**Measuraments and main results:**

Serum levels of procalcitonin, pro-adrenomedullin and pro-atrial natriuretic peptide were determined immediately after bypass and at 24–36 hours. Their values were correlated with the need for mechanical ventilation, inotropic support and bacterial infection. One hundred eleven patients were recruited. Septal defects (30.6%) and cardiac valve disease (17.1%) were the most frequent pathologies. 40.7% required mechanical ventilation, 94.6% inotropic support and 15.3% presented invasive bacterial infections. Pro-adrenomedullin and pro-atrial natriuretic peptide showed significant high values in patients needing mechanical ventilation. Cut-off values higher than 1.22 nmol/L and 215.3 pmol/L, respectively for each biomarker, may indicate need for mechanical ventilation with an AUC of 0.721 and 0.746 at admission and 0.738 and 0.753 at 24–36 hours, respectively but without statistical differences. Pro-adrenomedullin and procalcitonin showed statistically significant high values in patients with bacterial infections.

**Conclusions:**

After bypass, pro-adrenomedullin and pro-atrial natriuretic peptide are suitable biomarkers to predict the need for mechanical ventilation. Physicians should be alert if the values of these markers are high so as not to progress to early extubation. Procalcitonin is useful for predicting bacterial infection. This is a preliminary study and more clinical studies should be done to confirm the value of pro-adrenomedullin and pro-atrial natriuretic peptide as biomarkers after cardiopulmonary bypass.

## Introduction

Procalcitonin (PCT), pro-adrenomedullin (pro-ADM) and brain natriuretic peptide have been proposed as useful biomarkers for therapeutic decision-making in patients that require cardiopulmonary bypass (CPB), at both pre-and post-surgical time [1–5]. Recent studies underlined the promising utility of them as prognostic predictors in patients with heart failure or low cardiac output syndrome also in children [6–9]. PCT is known to be more specific and earlier than classical biomarkers in the diagnosis of infectious complications [10,11]. Although PCT may increase slightly in the systemic inflammatory response syndrome, as it occurs after CPB, most studies point to better predictive values of PCT than other clinical or analytical markers to discriminate the presence or absence of infection [6,12–14]. Other biomarkers such as pro-ADM and pro-atrial natriuretic peptide (pro-ANP) would be more appropriate in the stratification of the severity of critically-ill patients. These biomarkers can be even better prognostic markers than the traditional severity scores such as the Pediatric Risk Score Mortality III (PRISM III) [15–17]. Adrenomedullin is a potent vasodilator that can act as a hormone or cytokine and plays a role in controlling pulmonary flow, migration of leukocytes and electrolyte balance. Pro-ADM is a peptide directly measuring the blood levels of adrenomedullin, but biochemically more stable and easier to determine. Several studies have shown the usefulness of pro-ADM as a marker of severity and prognosis in patients with respiratory infections, and sepsis [18–20]. Some studies have described increased levels of adrenomedullin after CPB, thus it could be useful in the post-operatory [21,22]. A more recent study has shown that pro-ADM could accurately detect pediatric heart failure [7]. The brain natriuretic peptide and the pro-ANP have been proposed as markers of heart dysfunction, and as prognostic markers for monitoring patients with heart failure, acute coronary syndromes and hypertension [7,8,23–25]. There are many publications about the usefulness of brain natriuretic peptide in children [26], but not about pro-ANP. An increase of pro-ANP has been described in septic patients, and its usefulness as a prognostic marker in the CPB has also been suggested [27,28].

As far as we know, the studies linking these biomarkers and cardiac pathologies are basically oriented to describe their levels in cardiac failure and, in a lesser extent, cardiac surgery. In any case, these studies are scarce and tackle different heart diseases. In children, the number of studies is even lower and there are no clear conclusions that could be helpful in clinical practice. One of the main concerns when managing patients with CPB is to predict mortality and morbidity to anticipate its handling. Morbidity in patients after CPB was classically considered as the length of stay (LOS), but intensive support requirements after CPB could be also interesting to be determined as morbidity. Our aims in this investigation were to assess in critically-ill patients after CPB: i) PCT, pro-ADM and pro-ANP levels in the first 36 hours post-CPB; ii) the prognostic usefulness of these biomarkers comparing with PRISM III, regarding the need for respiratory and hemodynamic supports, and LOS after CPB and iii) the usefulness of these biomarkers as predictors of bacterial infection.

## Materials and methods

A prospective and observational study (July 2012-October 2013) was conducted on patients attended in the Pediatric Intensive Care Unit (PICU) of a tertiary-care children’s hospital with 345 beds (18 PICU beds). Referral population: Catalonia, with ~7 million population and 1.2 million children under 18 years, this captured around 17% of all pediatric hospital admissions during the study period. The study was approved by the ethics committee of the hospital (CEIm Fundacion Sant Joan de Deu, Barcelona) and the institutional review board. Written informed consent was obtained from parents or the legal guardian of each child.

The inclusion criteria were patients admitted at PICU after CPB, aged between 1 month and 16 years, and who provided signed informed consent. Exclusion criteria were underlying disease (rheumatologic, systemic) and presence of primary or secondary immunodeficiency.

Serum levels of PCT and the mild regional peptide segment of the prohormones pro-ADM and pro-ANP [29,30] were determined by immunofluorescence by means of a Time-Resolved Amplified Cryptate Emission (TRACE) and a Kryptor analizer (B·R·A·H·M·S-Diagnostica GmbH, Henningsdorf, Germany). Limit detection values were 0.06 ng/mL for PCT, 0.23 nmol/L for pro-ADM, and 2.1 pmol/L for pro-ANP. Values were considered normal for a PCT value < 0.5 ng/ml, a pro-ADM value < 0.5 nmol/L, and pro-ANP value < 70 pmol/L. The three biomarkers were collected from the same blood sample (1 ml). Sampling times were immediately post-CPB (post-CPB1), and between the 24–36 hours post-CPB (post-CPB2). The clinicians were blinded to the biomarkers results during the investigation.

The clinical parameters recorded were age; gener; type of heart disease; surgery time including surgery total time, bypass time and cross clamp time; ultrafiltrate volume; surgery complexity assessed with STS-EACTS score [31], and patient severity by PRISM III. Morbidity was evaluated through the need for mechanical ventilation (MV) at PICU admission and at 48 hours, use of inotropes at admission, presence of renal failure, invasive bacterial infection (IBI) complication (sepsis, pneumonia, urinary tract infection, meningitis) and PICU LOS; Mortality was also registered. In case of suspected infection, cultures of the different biological samples were processed: blood sample, urinary culture, and tracheal aspirate or broncho alveolar lavage. Pneumonia was suspected due to clinical data (fever > 38°C, tachypnea, respiratory worsening), thorax X ray with persistent infiltrates and C reactive protein > 70 mg/L. IBI was confirmed by positive cultures. Sepsis diagnosis was defined according to the local protocol (positive culture and life-threatening organ dysfunction [32]). Diagnosis of nosocomial infection was performed according to the criteria of the Centre for Disease Control and Prevention [33].

Sample characteristics were expressed as counts and percentage for categorical variables, as mean ± standard deviations for normal continuous variables, or as a median and interquartile range (IQR) for non-parametric data. Differences in PCT, pro-ADM and pro-ANP values were compared according to the morbidity and mortality data, by student T test or Mann-Whitney U test. The correlation of time of MV and pro-ADM were assessed using Pearson’s correlation. To compare the predictive value of PCT, pro-ADM, and pro-ANP, receiver-operating characteristic (ROC) curves and the area under the curve (AUC) were assessed. Prognostic parameters such as cut-off, sensitivity, specificity, positive and negative predictive values were calculated. Mean of PRISM III punctuation was correlated with PCT, pro-ADM and pro-ANP levels at admittance by means of a linear regression analysis. All tests were two-tailed. Probability values less than 0.05 were considered significant. We used the SPSS20.0 statistical software package.

## Results

### Sample characteristics

One hundred and eleven patients were included in the study, the 53.1% were males, and the median age was 26 months (IQR 7 months—6.5 years). Heart disease diagnoses were: 34 septal defects (30.6%), 19 cardiac valve disease (17.1%), 13 atrioventricular canal defect (11.7%), 12 Fallot tetralogy (10.8%), 6 single ventricle (5.4%), 3 transposition of the great arteries (2.7%) and 14 other heart disease (12.6%). Mean surgery total time, bypass time and cross clamp time were: 150 minutes (IQR 125–190), 70 minutes (IQR 54–95) and 41 minutes (IQR 28.50–66). Median PRISM III score was 3 points (IQR 3–5). The most frequent STAT mortality category was category 2 (48, 43.2%).

At admission, a total of 48 patients (40.7%) required MV whereas the rest were extubated at operating room. The median days of MV were 0 days (IQR 0–2). Inotropic support with milrinone was required in 105 patients (94.6%), and 34 patients (30.6%) required milrinone with any other inotropic drug (dopamine or epinephrine). Complications due to IBI were diagnosed in 15.3% of patients, with a final diagnosis of sepsis (47.1%) and pneumonia (29.4%) as the main causes of infection. Renal failure was not detected in any patient. One patient died at 72 hours.

### Biomarker levels

The median levels of the different biomarkers were at post-CPB1: for PCT 0.31 ng/ml (IQR 0.07–1.04); for pro-ADM 1.03 nmol/L (IQR 0.79–1.49); and for pro-ANP 174.90 pmol/L (IQR 106.60–339.40). Results at post CBP2 were: PCT 0.73 ng/ml (IQR 0.34–2.60); pro-ADM 1.13 nmol/L (IQR 0.81–1.83); and pro-ANP 147.10 pmol/L (IQR 95.16–285.70). Correlation values of the three biomarkers and several cardiac surgery characteristics are summarized in supplementary material 1. At post-CPB1, both pro-ADM and pro-ANP showed a significant positive correlation with bypass time and cross clamp time, and a significant negative correlation with ultrafiltrate. Pro-ADM also showed a significant correlation with surgery total time and PCT did not show any significant correlation. PRISM III value failed to demonstrate a significant correlation neither with surgery time nor with ultrafiltrate.

### Biomarkers and intensive support in PICU

**Fig 1:**
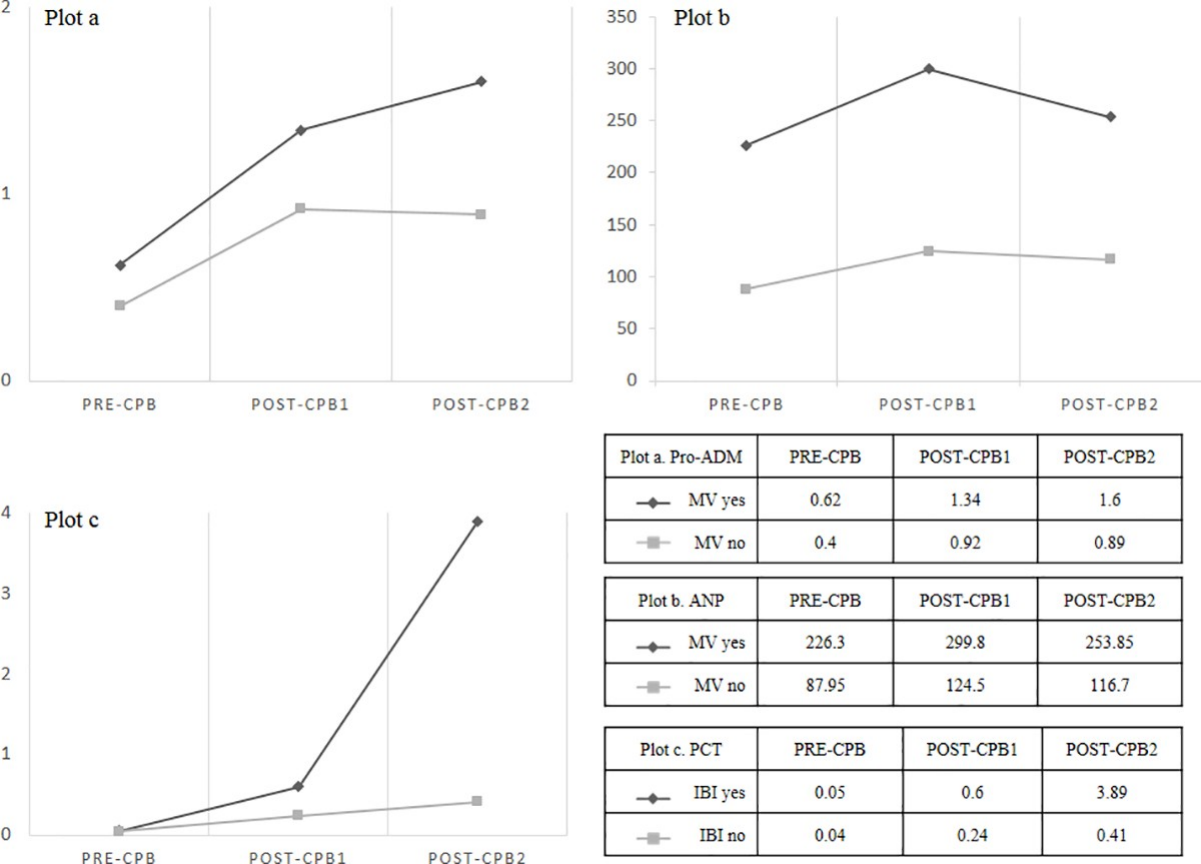
**Evolution of the median levels of pro-adrenomedullin (plot a) and pro-atrial natriuretic peptide (plot b) regarding mechanical ventilation (MV); and procalcitonin (plot c) regarding invasive bacterial infection (IBI) at pre and post cardiopulmonary bypass (CPB).** Values of pro-adrenomedullin (pro-ADM) expressed as nmol/L, pro-atrial natriuretic peptide (ANP) as pmol/L, values of procalcitonin (PCT) expressed as ng/mL.

The comparison between biomarker levels according to MV, IS and IBI are recorded in Table 1. Pro-ADM and PRO-ANP showed significantly higher values in those patients requiring MV or inotropic support with two drugs at post-CPB1 and post-CPB2. Pro-ADM at post-CPB1 and post-CPB2 presented positive correlations with the number of days of MV (*r* = 0.643 at post-CPB1 and *r* = 0.798 at post-CPB2, with *p*<0.001). Also PRO-ANP showed positive correlations with the days of MV (*r* = 0.557 at post-CPB1 and *r* = 0.631 at post-CPB2, with *p*<0.001). PCT values did not show significant differences in these comparisons. PCT and pro-ADM showed significant higher values in patients with IBI, both at post-CPB1 and post-CPB2 whereas PRO-ANP only showed significant higher values at post-CPB2. The evolution of the median levels of biomarkers regarding MV requirement for pro-ADM and PRO-ANP, or IBI complication for PCT is represented in Fig 1.

**Table 1:** Biomarkers levels at post-CPB1 and post-CPB2 according to mechanical ventilation, inotropic support and invasive bacterial infection. Data expressed by median (interquartile range) and compared with Mann-Whitney test.

	**post-CPB1**			**post-CPB2**		
**Biomarker levels**	**MV n = 48**	**Non-MV n = 63**	***p-value***	**MV n = 38**	**Non-MV n = 73**	***p-value***
**PCT** (ng/ml)	0.37 (0.06–1.20)	0.26 (0.09–0.80)	0.782	0.23 (0.06–1.16)	0.34 (0.13–0.88)	0.580
**Pro-ADM** (nmol/L)	1.35 (0.99–2.02)	0.92 (0.73–1.16)	0.000	1.34 (0.98–1.94)	0.91 (0.75–1.15)	0.000
**Pro-ANP** (pmol/L)	299.80 (147.10–476.58)	124.50 (89.25–230.05)	0.000	301.02 (144.95–464.45)	124.50 (86.97–221.30)	0.000
**Biomarker levels**	**Inotropic support** **n = 34**	**Non-inotropic support n = 77**	***p-value***	**Inotropic support n = 28**	**Non-inotropic support n = 83**	***p-value***
**PCT** (ng/ml)	0.42 (0.07–3.10)	0.28 (0.23–0.79)	0.283	0.38 (0.70–2.68)	0.31 (0.66–0.80)	0.590
**Pro-ADM** (nmol/L)	1.36 (0.99–2.69)	0.96 (0.74–1.27)	0.000	1.50 (1.03–2.70)	0.96 (0.74–1.25)	0.000
**Pro-ANP** (pmol/L)	282.25 (110.67–403.95)	146.40 (98.65–270.20)	0.010	318.55 (170.90–513.07)	140.45 (95.45–241.50)	0.000
**Biomarker levels**	**IBI** **n = 17**	**Non-IBI** **n = 94**	***p-value***	**IBI** **n = 17**	**Non-IBI n = 94**	***p-value***
**PCT** (ng/ml)	1.98 (0.275–5.53)	0.23 (0.07–0.75)	0.020	3.89 (2.03–6.00)	0.41 (0.071–0.63)	0.000
**Pro-ADM** (nmol/L)	1.44 (0.94–2.21)	1 (0.78–1.45)	0.036	1.84 (1.23–2.68)	0.95 (0.75–1.15)	0.000
**Pro-ANP** (pmol/L)	186.10 (137.70–350.02)	174.90 (104.55–341.30)	0.502	244.20 (138.95–507.7)	148.80 (92.97–326.05)	0.000

MV: mechanical ventilation, IBI: invasive bacterial infection, PCT: procalcitonin, pro-ADM: pro-adrenomedulin, Pro-ANP: pro-atrial natriuretic peptide, CPB: cardiopulmonary bypass.

Surgery times were significantly higher in patients with MV: Surgery total times were 170 minutes (IQR 140–235) vs. 138.5 (IQR 116.25–163.75), *p* = 0.000; bypass time 90 minutes (IQR 67–135) vs. 59.5 (IQR 47.25–77.75), *p* = 0.000; and cross clamp time 55 minutes (IQR 39.5–83.5) vs. 34.5 (IQR 22.5–77.45), *p* = 0.006. There were no statistical differences regarding ultrafiltrate. None of these surgery times showed significant differences either with inotropic support requirement or with IBI complications.

At post-CPB1, PRISM III showed significant higher values in patients with MV (score of 3, IQR 2–7) than in patients that do not required MV (score of 2, IQR 2–5). No statistical differences between PRISM III and MV were detected at post-CPB2. Similarly, PRISM III failed to show significant differences between patients according to inotropic support requirement or IBI complications.

Given the significant results of pro-ADM and pro-ANP regarding MV requirement, the cut-off points of these two markers were analyzed and are summarized in Table 2. Although pro-ANP showed slightly higher AUC values than pro-ADM at post-CPB1 (0.746 vs. 0.721) and post-CPB2 (0.753 vs. 0.738), no significant differences between AUCs for pro-ANP and pro-ADM were found. AUC for PRISM III to predict MV at post-CPB1 was 0.60 (CI95% 0.497–0.697*; p* = 0.0749) and the optimal cut-off was up to 2 points. The AUCs for pro-ADM and pro-ANP were considerably higher than the AUC for PRISM III but only the comparison between pro-ADM and PRISM III AUCs was statistically significant (*p* = 0.034). The AUC for bypass time and cross clamp time were higher to predict MV after CPB, than the surgical time and the ultrafiltrate volume (Table 2).

**Table 2:** Area under the curve for pro-ADM and pro-ANP measured at post-CPB1 and post-CPB2, and PRISM III value at post-CPB1 with regard to mechanical ventilation requirement.

Mechanical ventilation requirement							
Variables	AUC	*p* (CI 95%)	Cut-off	Sn % (CI 95%)	Sp % (CI 95%)	PPV % (CI 95%)	NPV % (CI 95%)
Pro-ADM CPB1	0.721	0.006 (0.568–0.845)	> 1.223	69.50 (36.40–79.30)	86.96 (76.44–97.20)	80.21 (54.16–95.17)	78.94 (60.24–91.60)
Pro-ANP CPB1	0.746	0.002 (0.589–0.881)	> 215.3	69.55 (32.20–75.63)	78.26 (56.35–92.55)	70.87 (46.61–88.69)	77.16 (57.07–90.98)
PRISM III	0.600	0.075 (0.497–0.697)	> 2	65.9 (50.77–79.12)	46.1 (32.20–60.53)	48.23 (29.08–67.77)	64.06 (39.21–84.37)
Surgical time	0.697	0.003 (0.576–0.800)	>160	62.9 (42.4–80.6)	75.0 (59.7–86.8)	60.7 (40.6–78.5)	76.7 (61.2–88.4)
Bypass time	0.767	<0.001 (0.680–0.841)	>80	63.8 (48.5–77.3)	79.7 (68.3–88.4)	68.2 (52.4–81.4)	76.4 (64.9–85.6)
Cross clamp time	0.776	<0.001 (0.687–0.849)	>47	66.7 (51–80)	76.1 (64.1–85.7)	65.2 (49.8–78.6)	77.3 (65.3–86.7)
Ultrafiltrate volume	0.538	0.517 (0.435–0.639)	<450	61.5 (44.6–76.6)	50 (36.8–63.2)	44.4 (30.9–58.6)	66.7 (50.9–80.1)
Pro-ADM CPB2	0.738	<0.001 (0.642–0.820)	> 1.01	72.92 (58.20–84.72)	64.81 (50.68–77.35)	51.85 (27.57–75.51)	82.16 (58.04–95.63)
Pro-ANP CPB2	0.753	<0.001 (0.658–0.833)	> 172.5	70.83 (55.91–83.02)	62.96 (48.74–75.71)	49.84 (25.89–73.85)	80.59 (56.26–94.83)

MV: mechanical ventilation, CPB: cardiopulmonary bypass, pro-ADM: pro-adrenomedullin, pro-ANP: pro-atrial natriuretic peptide, PRISM III: Pediatric Risk Score Mortality III, AUC: area under the curve, CI: confidence interval, Sn: sensitivity, Sp: specificity, PPV: positive predictive value, NPV: negative predictive value.

**Fig 2:**
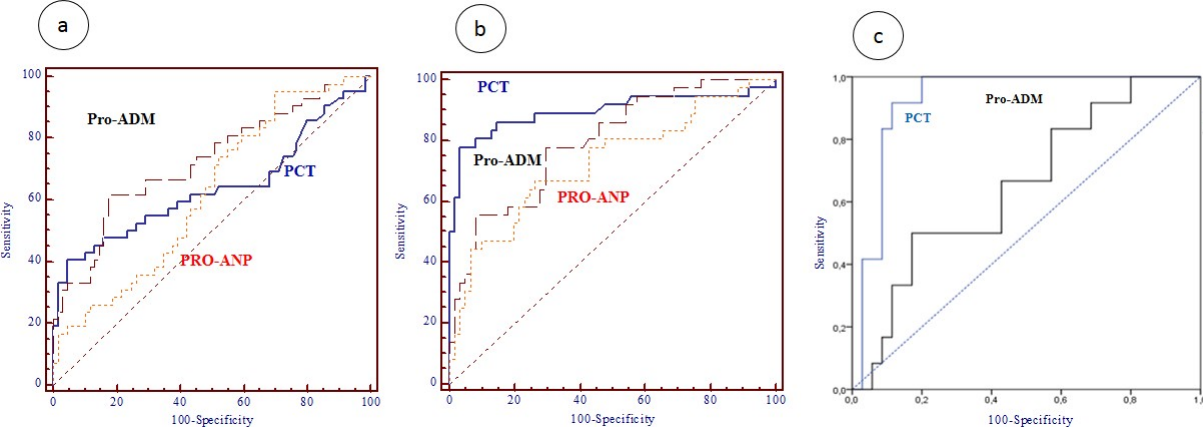
Areas under the curve for procalcitonin, pro-adrenomedullin and pro-atrial natriuretic peptide at post-CPB1 (plot a), at post-CPB2 (plot b). Plot c represents area under the curve for procalcitonin and pro-adrenomedullin at post-CPB2 (plot c).

When IBI was diagnosed, PCT, pro-ADM and pro-ANP levels were increased at both post-CPB1 and post-CPB2 (Table 1). The AUCs of the three biomarkers for this complication are reported in Table 3 and plotted in Fig 2. At post-CPB1, the AUC for pro-ADM (0.726) was higher than the AUCs for PCT (0.643) and pro-ANP (0.622). At post-CPB2, the biomarker with the highest AUC was PCT (0.896) followed by pro-ADM (0.795) and pro-ANP (0.722). When the AUCs between biomarkers were compared two by two, the only significant difference was found between PCT and pro-ADM at post-CPB2 (*p* = 0.013, plot *c* in Fig 2).

**Table 3:** Area under the curve (AUC) for the three biomarkers with respect to the presence of invasive bacterial infection. Data expressed by median (interquartile rang).

**post-CPB1**							
**Biomarker**	**AUC**	***p* (CI 95%)**	**Cut off**	**Sn % (CI 95%)**	**Sp % (CI 95%)**	**PPV (%)**	**NPV (%)**
**PCT** (ng/ml)	0.643	0.017 (0.549–0.729)	> 0.1	69.70 (53.9–82.8)	41.89 (30.5–53.9)	17.80 (1.83–53.32)	96.28 (40.83–99.93)
**Pro-ADM** (nmol/L)	0.726	<0.001 (0.633–0.806)	> 0.90	80.95 (65.9–91.4)	47.83 (35.6–60.2)	21.89 (2.70–59.69)	93.29 (50.43–99.99)
**Pro-ANP** (pmol/L)	0.622	0.023 (0.525–0.712)	> 140	71.43 (55.4–84.3)	49.28 (37.0–61.6)	20.28 (2.19–58.09)	90.52 (47.25–99.95)
**post-CPB2**							
**Biomarker**	**AUC**	***p* (CI 95%)**	**Cut off**	**Sn % (CI 95%)**	**Sp % (CI 95%)**	**PPV (%)**	**NPV (%)**
**PCT** (ng/ml)	0.896	0.001 (0.821–0.947)	> 1.9	78.95 (62.7–90.4)	95.52 (87.5–99.1)	79.09 (6.40–99.98)	96.17 (70.78–99.99)
**Pro-ADM** (nmol/L)	0.795	<0.001 (0.701–0.870)	> 1.1	77.78 (60.8–89.9)	63.93 (50.6–75.8)	28.03 (3.49–70.51)	94.09 (58.10–99.99)
**Pro-ANP** (pmol/L)	0.727	< 0.001 (0.627–0.812)	> 140.0	71.43 (55.4–84.3)	47.83 (35.6–60.2)	19.83 (2.06–57.64)	90.26 (46.96–99.95)

CPB: cardiopulmonary bypass, AUC: area under the curve, CI: confidence interval, Sn: sensibility, Sp: specificity, PPV: positive predictive value, NPV: negative predictive value, PCT: procalcitonin, pro-ADM: pro-adrenomedullin, Pro-ANP: pro-atrial natriuretic peptide.

Biomarker levels according to PICU LOS are reported in Table 4. As expected, levels were higher in those patients with more days of PICU stay but only the differences for pro-ADM and pro-ANP were statistically significant.

**Table 4:** Summary of the biomarker levels according to the length of stay in the pediatric critical care unit at post-CPB1. Date represented as median (interquartile range) and compared with Mann-Whitney test.

Biomarker levels	> 3 days PICU Yes (n = 42) vs No (n = 21)	*p*-value	> 7 days PICU Yes (n = 24) vs No (n = 37)	*p-*value
**PCT**(ng/ml)	2.23 (0.92–5.50) 2.10 (0.51–5.12)	0.973	2.40 (0.99–6.20) 2.02 (0.53–5.84)	0.653
**Pro-ADM** (nmol/L)	2.01 (0.80–2.98) 1.09 (0.45–1.60)	0.000	2.04 (0.85–2.99) 0.99 (0.1–0.68)	0.000
**Pro-ANP** (pmol/L)	387.35 (220.13–390.25) 185.33 (136.10–208.23)	0.009	440.99 (223.45–392.00) 180.83 (134.20–202.46)	0.018

PICU: pediatric intensive care unit; PCT: procalcitonin, pro-ADM: pro-adrenomedullin, pro-ANP: pro-atrial natriuretic peptide.

## Discussion

This study provides important information on the values of three biomarkers during the first 36 hours post-CPB. The results obtained underline for the first time the prognostic usefulness of pro-ADM and pro-ANP to predict need for prolonged MV and LOS after CPB on one hand, and confirm the value of the PCT to predict the infectious complication. The three biomarkers showed persistently high values in those patients that needed MV or inotropic support after surgery, but only the results of pro-ADM and pro-ANP were statistically significant at both times of the analysis. Pro-ADM and pro-ANP can be analysed with the same test in a few minutes (30–60 minutes), which provides valuable information for clinicians. In those patients with bacterial infection, pro-ADM and PCT showed statistically significant high values at post-CPB1 and post-CPB2. This finding could be due to the fact that pro-ADM and pro-ANP have a more important role in vasoregulation than PCT, which seems to be mainly activated by an infectious stimulus. During CPB there is a syndrome of systemic anti-inflammatory response with pathophysiological changes affecting homeostasis. There is an activation of the renin-aldosterone system increasing vascular resistances, diminishing urinary volume and presenting a third space due to fluid retention [34,35]. These changes may induce an increased production of ADM and pro-ANP to counteract the effects above mentioned and protect the heart muscle.

With regard to ADM and ANP, some studies demonstrated the progressive increasing of these biomarkers and their vasoregulatory role in CPB [35,36]. Our results are similar to those of Brancaccio and collaborators [37] in adults, describing an increase of ANP after CPB in response to the hypothermia and ischemia with an effect of myocardial protection. Nagata et al. described a correlation between cross clamp time and increase of ADM in 10 adults undergoing CPB [38]. The correlation between cross clamp time and increase of ADM suggests a vasodilator effect at coronary level to avoid ischemia. Szekely et al. showed that high levels of preoperative ADM are associated with less myocardial damage underlining the regulatory role of ADM in coronary flow both in ischemia and basal conditions [22]. The study of Hiramatsu showed an increase of ANP after Fontan procedure suggesting that ANP plays a mechanism of control of body fluids avoiding ascites and massive pleural effusion [39]. However, the regulatory role of pro-ANP in CPB is not yet well understood. There are publications regarding the usefulness of the brain natriuretic peptide in children after cardiac surgery [26], but there are still no data about the usefulness of pro-ANP in these patients. Brain natriuretic peptide and pro-ANP have a good correlation [40] and there exists the possibility to analyse pro-ANP and pro-ADM together in less than 30 minutes, that supposes an advantage. Also, pro-ANP could provide additional information in the BNP grey zone and in patients with obesity, as it was shown in adults [40].

According to our results, cut-off values of pro-ADM and pro-ANP higher than 1.223 nmol/L and 215.3 pmol/L respectively may indicate a greater affectation of the patient, with greater need for MV. Thus, early extubation of those patients would have to be carefully evaluated. In CPB, the classical parameters of early extubation have been surgical times, surgical difficulty and patients’ severity measured through risk scores such as PRISM III [41–45]. In our study, both parameters were higher in patients who required MV at admission than in those who did not. However, the AUC was higher for pro-ADM and pro-ANP than for surgical times or PRISM. In addition, the fact that PRISM III did not correlate either with biomarkers or surgical times could suggest that this score is influenced by other data not directly related to cardiac surgery. There are no previous studies that analyse pro-ADM or pro-ANP as useful tools to determinate a safe early extubation in post-CPB, but our results could be suggestive of that.

The incidence of IBI in children after CPB in this study is similar to previous published studies [46–49]. PCT was more useful as a diagnostic biomarker of infectious complication than as a prognostic biomarker. PCT cut-off values were better after 24 hours, as has already been observed in other studies [12,50,51]. The AUCs were similar to our previous study [50] and other works such as the one conducted by Aoufi [12]. In that study, a cut-off point for PCT above 1 ng/ml was a predictor of bacterial infection, and above 10 ng/ml for septic shock. Furthermore, Jebali found a significant PCT increase in infection cases even at the second post-operative day [52].

This study has some limitations that should be stated. Firstly, the total number of patients is relatively low and the results regarding the type of heart disease and type of surgery have not been analysed, thus it is not possible to know whether the type of surgery may have any influence. Secondly, this study should be considered as preliminary because of the lack of similar studies. In view of our results, it would be interesting to carry out a clinical study to confirm and extent the pro-ADM and pro-ANP value as markers of need for MV after CPB. An analysis for sub-groups of congenital heart diseases would be valuable to detect possible confounding factors. If they are suitable markers, biomarker determinations should be done immediately after CPB in order to decide the best moment for patient extubation, as a practical tool.

## Conclusions

The persistently high values of pro-ADM and pro-ANP after CPB in those patients that needed MV or inotropic support underline their prognostic usefulness. Pro-ADM and pro-ANP are good predictors of need for MV and LOS after CPB. A high value of these biomarkers at admission in PICU after CPB should alert the intensivist not to proceed to an early extubation. Further investigations are needed to enhance our knowledge about these biomarkers.
